# Gastric villous adenoma: a case report and review of the literature

**DOI:** 10.1186/s13256-023-03893-2

**Published:** 2023-04-26

**Authors:** Phalat Sathirawich, Ananya Pongpaibul, Thammawat Parakonthun, Uayporn Kaosombatwattana

**Affiliations:** 1 Division of Gastroenterology, Department of Medicine, Faculty of Medicine Siriraj Hospital, Mahidol University, 2 Wanglang Rd. Siriraj Subdistrict, Bangkok-noi, Bangkok, 10700 Thailand; 2 Vikit Viranuvatti Siriraj GI Endoscopy Center, Siriraj Hospital, Mahidol University, Bangkok, Thailand; 3 Department of Pathology, Faculty of Medicine Siriraj Hospital, Mahidol University, Bangkok, Thailand; 4 Division of Minimally Invasive Surgery, Department of Surgery, Faculty of Medicine Siriraj Hospital, Mahidol University, Bangkok, Thailand

**Keywords:** Villous adenoma, Gastric polyp, Adenomatous polyp

## Abstract

**Background:**

Villous adenoma is the one subtype of adenomatous polyp that is very uncommon in the stomach. Data regarding clinical characteristics, natural history, and prognosis were scarce.

**Case presentation:**

This report presented an 87-year-old Thai woman with a large gastric villous adenoma incidentally revealed in a computed tomography of chest for the evaluation of right pleural effusion. The esophagogastroduodenoscopy demonstrated a huge, glossy, proliferative polypoid mass involving gastric cardia, fundus, and a lesser curve of the upper body. The pathological report confirmed villous adenoma with low grade dysplasia. Although surgical resection was suggested, the patient denied any treatment due to advanced age and multiple comorbidities. She was generally well after 12 months of clinical and radiologic surveillance.

**Conclusion:**

From literature review, only 14 cases of gastric villous adenoma were reported to date. Most of the lesions were large and symptomatic. Malignancy presented in 43% of the cases. Nevertheless, our patient remained asymptomatic without surgical removal following a 12-month period.

## Introduction

Adenomatous neoplasm is commonly observed in the colorectum, and categorized as tubular, tubulovillous, and villous adenoma. The villous adenoma is the least common pathological finding among the adenomatous lesions [1]. Gastric polyp usually presents as an incidental finding during upper endoscopy, and fundic gland polyp is the most common pathology [2, 3]. Gastric adenomatous neoplasm is infrequent, and the gastric villous adenoma is exceedingly rare. Patients with sizeable gastric polyp can present with abdominal pain, upper gastrointestinal bleeding, and obstruction [4]. Less is known about the risk of malignancy and treatment outcome. Our report presents the case of a large gastric villous adenoma with long-term, clinical follow-up, including a review of the literature.

## Case report

**Fig. 1:**
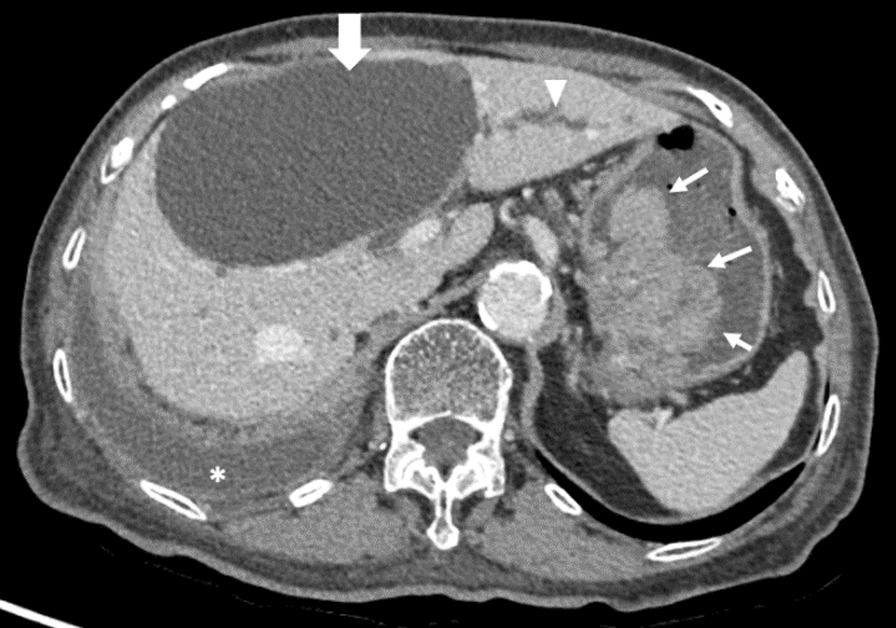
Venous phase of computed tomography at upper abdomen level. A 6.9 × 4.0 × 3.4 cm enhancing polypoid lesion in the stomach involving lesser curvature and gastric fundus (thin arrow), a large hepatic cyst (thick arrow) with adjacent intrahepatic duct dilation (arrow head), and right pleural effusion (*) were demonstrated

An 87-year-old Thai woman was diagnosed with hypertension, hyperlipidemia, gout, hypothyroidism, and completely cured right breast cancer for 30 years. The lymphatic obstruction from prior right radical mastectomy resulted in loculated right chylothorax for 1 year. The computed tomography (CT) of chest for evaluation of chylothorax incidentally revealed a huge polypoid mass involving lesser curvature of the stomach. Moreover, a large hepatic cyst at segment 4 with compression to the adjacent biliary tract was also observed (Fig. 1). Her hemoglobin was stable at 10 g per deciliter (g/dL) and her liver biochemical test was unremarkable. At that time, esophagogastroduodenoscopy was not proceeded with given her massive right pleural effusion.

She visited an emergency department 5 months later with acute right upper quadrant abdominal pain, and CT whole abdomen was performed. The size and characteristics of the hepatic cyst and biliary tract dilatation were unchanged. However, inferior vena cava thrombus was newly observed. The enhancing lobulated polypoid mass in the proximal stomach was again noted without significant change in size. Blood test remained unchanged, and she neither experience jaundice nor overt gastrointestinal bleeding. Eventually, the discussion with the patient and relatives for evaluation of gastric mass led to the esophagogastroduodenoscopy (EGD).

**Fig. 2:**
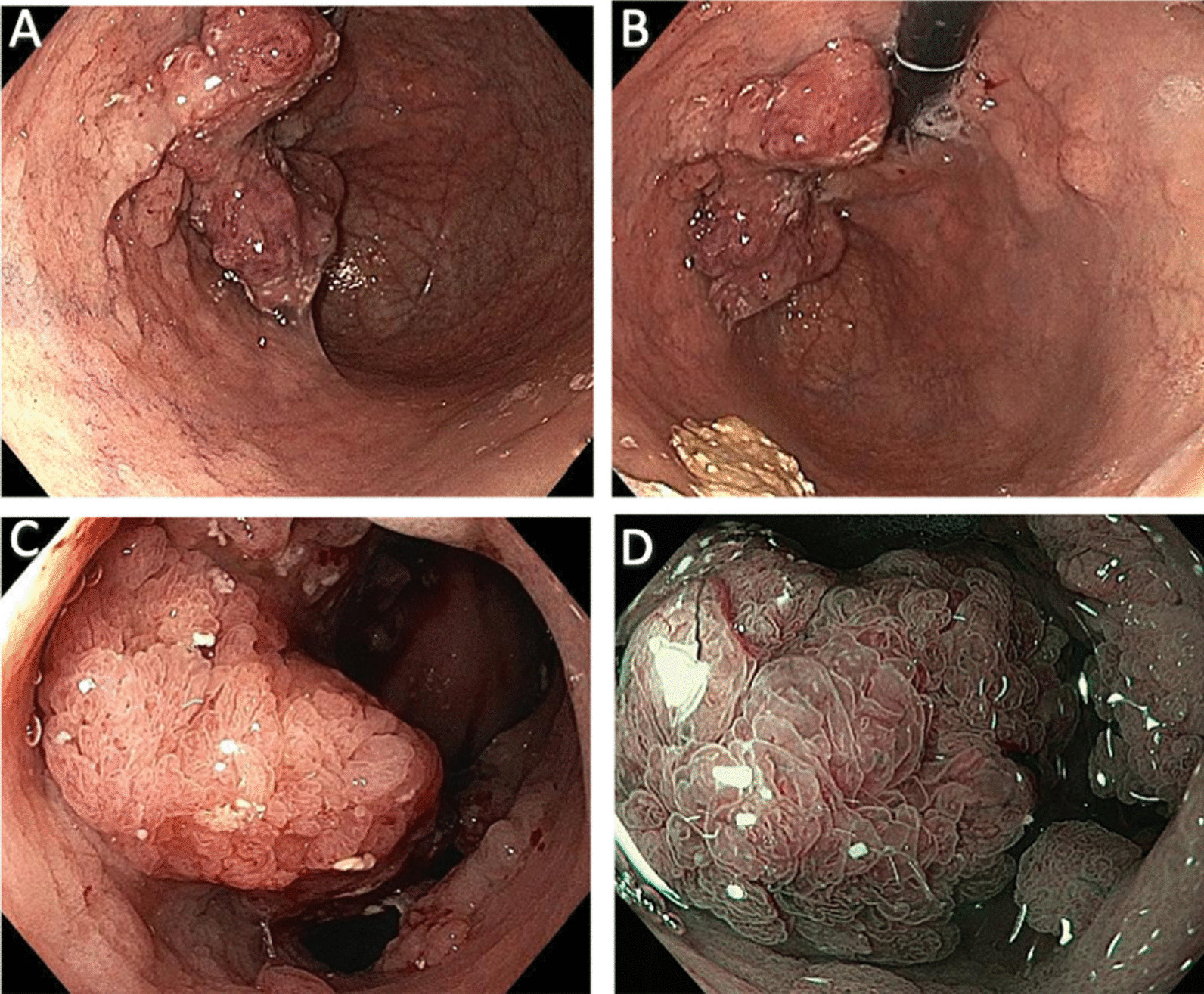
Esophagogastroduodenoscopy. White light image showed a large polypoid mass involving the gastric fundus and upper body in retroflexion view (**A**, **B**) and forward view (**C**). Image-enhanced endoscopy with narrow-band imaging showed papillary mucosal appearance with regular vascular pattern (**D**)

**Fig. 3:**
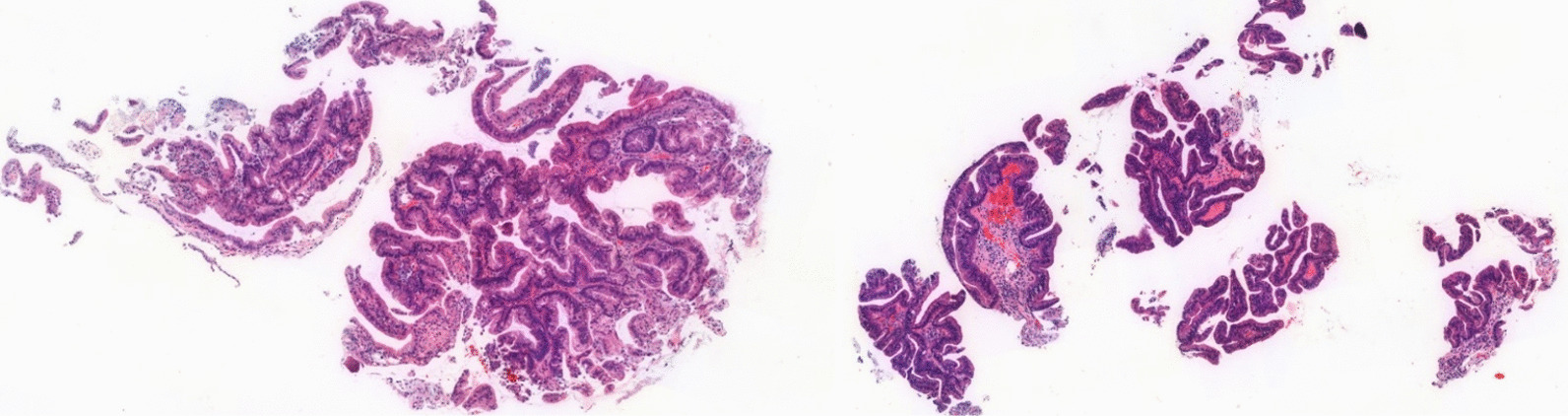
Gastric mucosa revealed chronic active gastritis with intestinal metaplasia and low-grade dysplasia (H&E staining, 4×)

A subsequent EGD demonstrated a large polypoid mass (0-Is) involving gastric cardia, posterior wall of the fundus, and a lesser curve of the upper body. The maximum diameter was around 7 × 4 cm. The mucosal surface contained a glossy, proliferative papillary appearance. The image-enhanced endoscopy with narrow-band imaging showed a regular vascular pattern without an area of vascular disappearance (Fig. 2). Microscopic examination revealed moderate chronic active inflammation with focal intestinal metaplasia. Foveolar epithelium showed villiform change with elongated hyperchromatic nuclei (low grade dysplasia). Numerous* Helicobacter pylori* were presented on the surface (Fig. 3).

**Fig. 4:**
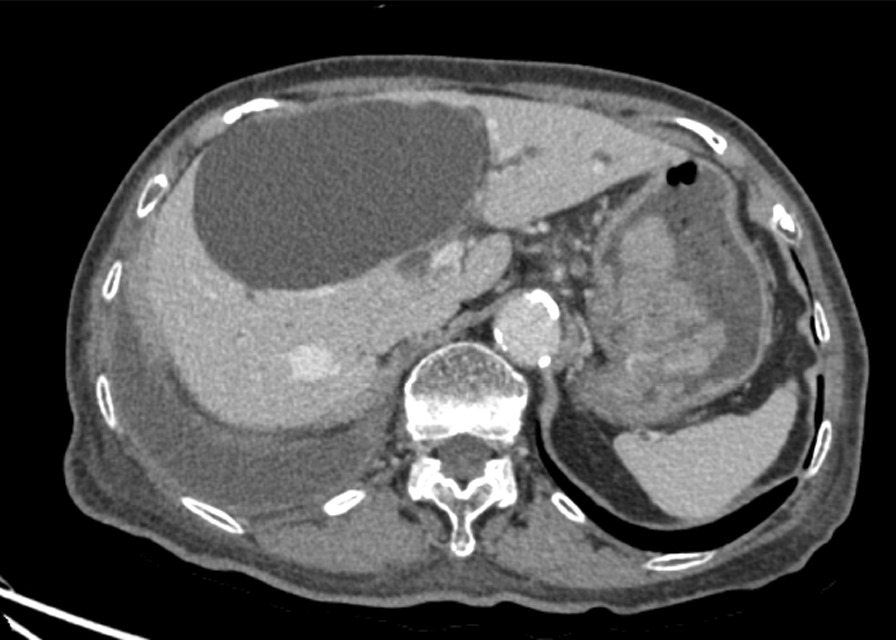
One-year interval of contrast-enhanced abdominal imaging demonstrated non-significant changing in size of enhancing polypoid mass lesion in the stomach involving lesser curvature

According to the bulky size of the polyp, endoscopic resection was not considered. Surgical removal was proposed to the patient and her relatives. Nevertheless, her advanced age and multiple comorbidities precluded her from the operation. The clinical symptoms were followed for 12 months, and she continues to have generally well condition without overt bleeding, anorexia, weight loss, or abdominal pain. Her hemoglobin remained at 11 g/dl with an iron supplement. No serum electrolyte disturbance had been observed. CT abdomen showed no significant changing in size of the gastric polyp (Fig. 4).

## Discussion

Gastric polyp is observed in approximately 2% of all upper gastrointestinal endoscopies. The adenomatous polyp was less familiar, with prevalence of 3.6% in overall gastric polyps [5]. Its prevalence increases parallel with age, from 0.1% in the third decade of life to 3.7% in the ninth decade [6]. According to pathological appearance, gastric adenomatous polyps were classified into three subtypes: tubular, tubulovillous, and villous adenoma. They are usually found in a solitary lesion, antral predominance, atrophic gastritis, and intestinal metaplasia background [2, 7].

Unlike colorectal villous adenoma, gastric villous adenoma was considered an extremely rare subtype of gastric neoplastic polyp. Its rarity comes along with the highest rate of malignant transformation of 29–40%, while only a 5% rate of malignant transformation was observed with gastric tubular adenoma [8]. The macroscopic characterization of gastric villous adenoma revealed a soft, polypoid epithelial mass with long papillary projections and intervening deep fissuring [9]. Microscopic appearance showed finger-like projection with villous features more than 75% of the polyp. As the appearance of papillary projection, the radiologic finding of “soap bubble” effect and frondlike mass lesion can be demonstrated from the upper gastrointestinal series and computed tomographic studies, respectively [10, 11].

The presenting symptoms of gastric villous adenoma were nonspecific, including fatigue, abdominal pain, upper gastrointestinal (GI) bleeding, or anemia without overt bleeding [10, 12]. The presence of voluminous watery diarrhea and the loss of potassium from secretory effect of the polyp, which was reported in the colorectal villous adenoma, is less frequent in the gastric villous adenoma [13]. Only one case was reported with severe electrolyte imbalance in large gastric villous adenoma [14]. This is probably due to the absorptive potential of the rest of the entire bowel. An extensive literature search from inception to July 2022 on clinical data of gastric villous adenoma only yielded reports of 14 cases from 9 case series (Table 1) [9–12, 15–19]. The mean age of these patients was 67 years, with a slight female predilection. Tumor size ranged from 2 cm to 21 cm, resulting in epigastric pain, vomiting, anemia, and GI blood loss. All but one patient underwent successful polyp resection, and malignancy presented in 43%.

**Table 1:** Previous case series of gastric villous adenoma

Case no. (Ref.)	Gender	Age	Presenting symptoms	Hb (g/dl)	Size (cm)	Presence of malignancy	Resection	Follow-up time (years)
1 [15]	M	62	Anemia	NA	9 × 5	No	Yes	1.5
2 [15]	F	69	Referral to the heart	NA	5 × 4	No	Yes	2 days^a^
3 [16]	F	62	Epigastric pain	NA	NA	Yes	Yes	NA
4 [11]	M	44	Epigastric pain, tarry stool	NA	11 × 15 × 4	Yes	Yes	3
5 [17]	F	72	Weight loss	11.7	15 × 13	Yes	Yes	NA
6 [18]	M	80	Postprandial vomiting	NA	4.5 × 3.5	Yes	Yes	6
7 [18]	F	56	Epigastric pain, vomiting	NA	3 × 2	No	Yes	NA
8 [19]	F	78	Anemia	6.4	21 × 15 × 10	No	Yes	2 months
9 [9]	F	59	Epigastric pain	NA	8 × 5 × 5	No	Yes	NA
10 [10]	F	73	Epigastric pain, weight loss	9.5	5	No	Endoscopic polypectomy	NA
11 [10]	M	70	Weight loss	11.3	NA	Yes	Yes	NA
12 [10]	F	75	Weight loss	6.8	NA	No	No^b^	NA
13 [10]	M	80	Anemia, weight loss	8.6	NA	Yes	Yes	NA
14 [12]	M	51	Hematemesis	9.5	NA	No	NA	NA

*F* female, *Hb* hemoglobin, *M* male, *NA* not applicable

^a^The patient died after an operation due to pulmonary embolism

^b^The patient refused further treatment

Management of gastric adenomatous polyp, including villous adenoma, is complete resection due to increased risk of malignant transformation. The British Society for Gastroenterology (BSG) guidelines for the management of gastric polyp recommend complete removal of all adenomatous polyps if the benefit outweighs the risk in operation together with the intensive whole gastric examination [8]. Likewise, the American Society for Gastrointestinal Endoscopy (ASGE) in management of the premalignant and malignant condition of the stomach suggested endoscopically removing all adenomatous polyps when possible [20]. Both guidelines recommended endoscopic surveillance in the 1-year interval after polyp removal. If resection is incomplete or high-grade dysplasia is present, surveillance should be performed 6 months after polyp removal, followed by follow-ups every 3–5 years [8, 20].

## Conclusion

Gastric villous adenoma is a rare gastric adenomatous polyp with a unique endoscopic and histological appearance. Here, we present a case of large gastric villous adenoma in an asymptomatic elderly patient. Even though it carries a high risk of malignant transformation, complete polyp removal was not performed. The patient remained asymptomatic during 12 months of clinical follow-up.

## Data Availability

The data that support the findings of this study are available from the corresponding author, upon reasonable request.
